# Presence of Terminal EPIYA Phosphorylation Motifs in *Helicobacter pylori* CagA Contributes to IL-8 Secretion, Irrespective of the Number of Repeats

**DOI:** 10.1371/journal.pone.0056291

**Published:** 2013-02-07

**Authors:** Konstantinos S. Papadakos, Ioanna S. Sougleri, Andreas F. Mentis, Efstathios Hatziloukas, Dionyssios N. Sgouras

**Affiliations:** 1 Laboratory of Medical Microbiology, Hellenic Pasteur Institute, Athens, Greece; 2 Laboratory of Molecular Biology, Department of Biological Applications and Technology, University of Ioannina, Ioannina, Greece; University of Hyderabad, India

## Abstract

CagA protein contributes to pro-inflammatory responses during *H. pylori* infection, following its intracellular delivery to gastric epithelial cells. Here, we report for the first time in an isogenic background, on the subtle role of CagA phosphorylation on terminal EPIYA-C motifs in the transcriptional activation and expression of IL-8. We utilized isogenic *H. pylori* mutants of P12 reference strain, expressing CagA with varying number of EPIYA-C motifs and the corresponding phosphorylation defective EPIFA-C motifs while preserving intact the CM multimerization motifs. These mutants had been previously closely scrutinized in terms of type IV secretion system functionality, CagA translocation and its subsequent phosphorylation. Following infection of gastric epithelial cell lines, transcriptional activation of IL-8 gene and secreted IL-8 levels were found to be strictly dependent upon the functionality of the EPIYA-C phosphorylation motifs, as EPIFA-C phosphorylation-deficient CagA expression failed to induce full IL-8 transcriptional activity. Interestingly, levels of IL-8 gene activation and of secreted IL-8 were the same, irrespective of the number of EPIYA-C terminal repeats. We monitored IkBα phosphorylation and confirmed CagA involvement in NF-kB activation. Furthermore, we observed that presence of EPIYA-C functional phosphorylation motifs contributed to NF-kB activation. NF-kB upstream signaling events, such as early ERK1/2 and AKT activation were confirmed to be independent of EPIYA-C phosphorylation. On the contrary, use of TAK1 specific inhibitor 5Z-7-Oxozeaenol resulted in complete arrest of IL-8 secretion, in a dose-dependent manner, irrespective of CagA status. *H. pylori*-infected TAK1^-/-^ mouse embryonic fibroblasts (MEFs) failed to induce NF-kB activity, unlike the respective control MEFs. CagA and TAK1 were found to immunoprecipitate together, irrespective of CagA EPIYA-C status, thus confirming earlier reports of TAK1 and CagA protein interaction. Our data suggest that CagA may potentially interfere with TAK1 activity during NF-kB activation for IL-8 induction in early *H. pylori* infection.

## Introduction

*Helicobacter pylori* (*H. pylori*) colonize the gastric mucosa of 35–70% of people worldwide and remain the main etiologic factor for development of chronic active gastritis and peptic ulcer [1], [2]. However, infection is usually asymptomatic in the vast majority of hosts, as virtually all carriers develop superficial chronic active gastritis, whereas only about 10% will suffer gastric or duodenal ulceration and 0.5–2% will develop gastric adenocarcinoma or B-cell lymphoma of mucosa-associated lymphoid tissue (MALT) [3]. The presence of neutrophil polymorphs in a background of chronic inflammation remains the hallmark of *H. pylori* infection, because neutrophil activity is an almost universal phenomenon in *H. pylori* gastritis [4]. Neutrophils can be found in biopsy specimens, in virtually all cases of *H. pylori*-positive patients and they are a very sensitive indicator of the presence or absence of *H. pylori* because they disappear within days of cure of infection [4], [5]. Interleukin 8 (IL-8), a CXC chemokine specific for neutrophil granulocyte chemotaxis, has been shown to be upregulated in *H. pylori* infected patients [5], [6] and to correlate with histological severity of gastritis [7]. Early reports have linked elevated inflammatory response and cytokine expression *in vivo,* to *cagA*-positive *H. pylori* strains [8]. CagA protein is a major *H. pylori* virulence factor which has gained much interest from a clinical point of view, as a marker of *H. pylori*-associated disease, having been shown to confer increased risk for development of atrophic gastritis, peptic ulcer, gastric cancer and lymphoma [1], [3], [9], [10]. From a mechanistic standpoint, CagA activity in gastric epithelial cells has been shown to be associated with disruption of intercellular junctions and of gastric epithelial cell polarity, increased cell motility and elongation cellular phenotypes, as well as propagation of signaling pathways relating to proliferation and inflammation in the gastric epithelium [11]. To this effect, CagA has been reported to interact with a number of intracellular key molecules, such as Shp2, Ras GTPase activating protein (Ras-GAP), phosphoinositide-3 (PI3)-kinase, and the adaptor proteins Crk, Grb2 and Grb7 [12]–[15].

The gene coding for CagA resides at the end of the cag Pathogenicity Island (cagPAI), a gene cluster of 40 Kbps that altogether codes for a type IV secretion system (T4SS), via which CagA, is translocated from the epithelium-adhered bacteria into gastric epithelial cells [16]. Early reports clearly correlate IL-8 induction in gastric epithelial cells to a functional cagPAI [17]. Once inside the cell, CagA is phosphorylated by host cellular kinases Src [18], [19] and Abl [20], [21], on repeating Glutamic Acid-Proline-Isoleucine-Tyrosine-Alanine (EPIYA) tyrosine phosphorylation motifs located at the carboxyl terminus of the protein. EPIYA motifs found in *H. pylori* strains of Western origin constitute three distinct types according to the surrounding aminoacid sequence, namely EPIYA-A (*EPIYA*KVNKKK(A/T/V/S)GQ), EPIYA-B (*EPIY*(*A/T*)(Q/K)VAKKVNAKI) and terminal EPIYA-C (*EPIYA*TIDDLG) [22]. These motifs vary considerably among strains regarding both the type and number, which may account for the differences observed in the pathogenic potential of *H. pylori* [23]. Hierarchic intracellular phosphorylation of CagA has been recently demonstrated, with c-Src only phosphorylating EPIYA-C motifs, whereas c-Abl phosphorylating EPIYA-A, EPIYA-B, and EPIYA-C [24]. Moreover, further analysis revealed that CagA molecules were phosphorylated on 1 or 2 EPIYA motifs, but never simultaneously on 3 motifs [24]. The number of EPIYA-C motifs has been suggested to determine the levels of CagA phosphorylation [25], to induce increasing levels *H. pylori*-related cytoskeletal rearrangements in epithelial cells *in vitro* [25], [26], and to confer oncogenic potential [22]. However, we have reported that in children [27] and in adult patients [28] the number of CagA EPIYA-C motifs does not correlate to more severe inflammatory response in the lamina propria. Moreover, in adults the presence of one CagA EPIYA-C site was found to be an independent risk factor for presence of gastro-duodenal ulceration [28].

In early studies involving *in vitro H. pylori* infection of gastric epithelial cells, proteins encoded by the cagPAI, with the exception of CagA, were shown to be required for IL-8 secretion and that IL-8 induction was regulated by the NF-kB pathway [9], [10]. In addition, *H. pylori* peptidoglycans delivered through the type IV secretion system, identified by intracellular Nod1 receptor were also reported responsible for activating NF-kB leading to IL-8 induction [29]. It was later that CagA protein, was also recognized to contribute to the induction of IL-8 in gastric epithelial cells, through the activation of NF-kB pathway [30]. Others suggested that IL-8 activation was not dependent upon CagA phosphorylation on EPIYA-C motifs but on the highly conserved amino acid sequence FPLKRHDKVDDLSK termed as CRPIA [31], earlier identified as CagA multimerization (CM) motif [32] which has since been identified as a MARK2-protein kinase inhibitor (MKI) [33]. CagA has also been suggested to stimulate NF-kB induction and transcriptional activation of IL-8, through interaction and subsequent activation of TAK1 and this was mediated by TRAF6-Lys 63-ubiquitination [34]. However, *H. pylori*-induced proinflammatory responses remain a controversial issue, due to emerging contradictory reports [35]. Very recently CagL, has been suggested to induce secretion of interleukin-8 (IL-8) independently of CagA translocation and peptidoglycan-sensing nucleotide-binding oligomerization domain 1 (NOD1) signaling [36].

The aim of this study was to investigate CagA involvement in the secretion of IL-8 and more specifically to explore the molecular pathway by which phosphorylation on CagA terminal EPIYA-C motifs could potentially contribute to IL-8 gene induction. For this purpose, and in order to study CagA expression inside the epithelial cells through an infection, rather than transfection system, a series of *H. pylori* isogenic mutants based on P12 reference strain were produced, expressing CagA protein with varying numbers of phosphorylation-functional (EPIYA-C) and phosphorylation-deficient EPIFA motifs, while keeping the CM motifs intact. These strains were meticulously evaluated beforehand for their ability to adhere equally well on gastric epithelial cells, induce pilus formation and functionally translocate CagA protein inside epithelial cells [37]. Utilizing these strains we were able to show that CagA EPIYA-C phosphorylation is required for full transcriptional activation of IL-8 gene, irrespective of the number of these repeating units at the carboxyl-terminus of CagA. Moreover, our data suggest that CagA protein may contribute to NF-kB activation, potentially through interference with TAK1 and independently of ERK1/2 and AKT mediated NF-kB activation.

## Materials and Methods

### H. pylori strains

**Figure 1:**
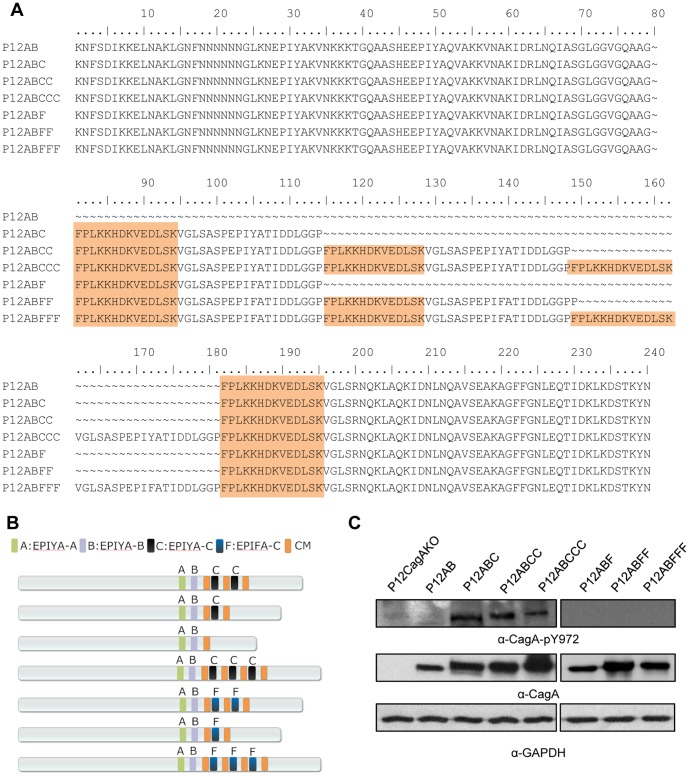
CagA EPIYA-C phosphorylation-functional and -defective *H. pylori* mutant strains. (A) Deduced CagA amino acid sequences, following nucleotide sequencing, depicting the EPIYA and EPIFA mutant motifs, as well as the MARK2-kinase inhibitor (CM) (shaded region). (B) Schematic representation of CagA protein expressed by the corresponding mutants. (C) Determination of CagA tyrosine phosphorylation by western blot utilizing α-CagA-pY972 antibody, which recognizes phosphorylated EPIYA-C motifs. CagA and GAPDH expression is also depicted, for control purposes.

Based upon *H. pylori* P12 reference strain, which possesses CagA with an EPIYA-ABCC combination, a number of isogenic *H. pylori* mutants expressing CagA protein with a variable number of EPIYA-C motifs such as AB, ABC, ABCCC and the respective phosphorylation-deficient EPIFA mutants (Figure 1A and Figure 1B) were constructed . A CagA knock out (P12CagAKO) strain was constructed by interruption of *cagA* gene sequence following insertion of a chloramphenicol cassette [38]. A T4SS-defective *cagE* knock-out mutant of P12 (P12CagEKO) was kindly provided by Professor R. Haas. These strains were meticulously characterized in terms of growth rates and adhesion rates to AGS gastric epithelial cells (ATCC CRL 1739), as well as T4SS functionality, CagA expression and phosphorylation and the ability to induce scattering and elongation phenotype[37].

### Culture of *H. pylori* and cell lines

*H. pylori* strains were cultured on Columbia Blood Agar (Oxoid) plates containing antibiotics, supplemented with 5% v/v horse blood and 1% v/v Vitox (Oxoid) under microaerophilic conditions (CampyGen, Oxoid) at 37°C, as described before [27].

AGS gastric epithelial cell line (ATCC CRL 1739) were cultured in 75 cm^2^ flasks (Corning) in RPMI 1640 medium (Life Technologies) containing antibiotics (penicillin 10 U/ml, streptomycin 10 mg/ml) and supplemented with 10% FBS (Life Technologies).

TAK1^-/-^ mouse embryonic fibroblasts (MEFs) and the respective control MEFs [39] were kindly provided by Professor S. Akira and were cultured as described in their publication.

### *In vitro* infection and protein immunodetection

Gastric epithelial cell lines (AGS) were infected with *H. pylori* at MOI 100. Briefly, 4×10^5^cells were seeded in 6-well plates and were left to adhere overnight. On the following day, two hours prior to infection, cells were washed with PBS (1x) and the medium was replaced with fresh, antibiotic free, RPMI 1640 containing 10%FBS. *H. pylori* strains were suspended in antibiotic free RPMI 1640 containing 10%FBS, adjusted to a concentration of approximately 10^8^cfu/ml (OD_600nm_ = 0.740) and left for an hour to recover. Bacterial suspensions (100 µl) were used to infect gastric epithelial cells within 2 ml total volume.vAt collection times, total protein lysates were obtained in ice-cold lysis RIPA buffer as described before [27]. Equal protein amounts of lysates were separated by SDS-PAGE and transferred onto PVDF membranes (Immobilon P, Merck Millipore). Antibodies against IKKa, phospho-IKBa (Ser32/36), phospho-ERK1/2 (Thr202/Tyr204), TAK1, phospho-TAK1 (Thr187), AKT, phospho-AKT (Ser473) (all purchased from Cell Signaling), CagA (polyclonal, Austral Biologicals) and GAPDH (Merck Millipore), were used according to suppliers' instructions. Specific antibody phospho-CagA (Tyr 972), which recognizes specifically phosphorylated CagA at EPIYA-C motifs, was kindly provided by Professor S. Backert [40]. Quantity One software package (Bio-Rad) was utilized for band densitometry.

### Immunoprecipitation

AGS cells (1×10^6^) were seeded in 25 cm^2^ flasks and were infected with *H. pylori* mutant strains at MOI 100. Following 1h of infection, cells were lysed by addition of 500 µl of NP40 lysis buffer containing protease and phosphatase inhibitors as described before [27]. Lysates were pre-cleared following incubation with 30 µl of Protein A-Sepharose from *Staphylococcus aureus* (Sigma). Respective immunoprecipitations were performed by addition of CagA polyclonal antibody (Austral Biologicals) or TAK1 monoclonal antibody (Cell Signaling) and consequent capture of the protein complex using 30 µl of Protein A-Sepharose. CagA detection by western blot was performed utilizing an anti-CagA monoclonal antibody supplied by Austral Biologicals or an anti-CagA monoclonal antibody raised against amino acids 1–300 supplied by Santa Cruz Biotechnology.

### Quantification of IL-8 gene expression in *H. pylori* infected cells

Quantification of IL-8 gene expression relative to GAPDH, in *H. pylori*-infected (MOI 100) and uninfected AGS cells, was determined by the comparative Ct method [41]. RNA samples were collected at 2, 4 and 24 hours post infection in triplicates using the RNeasy Mini Kit (Qiagen). DNA sample contamination was eliminated with the use of RNase-Free DNase Set (Qiagen). cDNA was synthesized withvM-MLV (Promega) reverse transcriptase (80U) in a reaction that contained 20 mM Tris (pH 8.4), 50 mM KCl, 3.75 mM MgCl_2_, 500 µM DNTPs, 0.4 µM, random hexamers primers (Life Technologies), 80 U RNase OUT Recombinant Ribonuclease Inhibitor (Life Technologies) and 22,2 µl RNA. Quantitative Real Time PCR amplification was carried out in a Stratagene Mx3005P QPCR System (Agilent Technologies), within 25 µl reaction mixtures of Platinum SYBR Green qPCR SuperMix (Life Technologies), 0.5 µM primers and 5 µl cDNA. Previously described PCR primers for human IL-8 [42], and GAPDH [43] were used. Three independent experiments were conducted involving three replicates for each individual sample time point.

### Determination of IL-8 levels and inhibitors

Culture supernatants collected at selected time points from *H. pylori*-infected AGS cells (MOI 100), were collected following centrifugation at 13,000 rpm [27]. IL-8 levels were determined by commercial ELISA (eBioscience) according to manufacturer’s protocol. Experiments in the presence of 5Z-7-Oxozeaenol (Merck) TAK1 specific inhibitor were carried out by addition of the inhibitor 2h prior to infection. Culture supernatants were collected at 4h post-infection. Initial experiments were conducted at a concentration range of 5–300 nM to determine the optimal concentration for TAK1 inhibition and then at 70nM thereof.

## Results

### Effect of EPIYA-C phosphorylation on IL-8 transcriptional activation and IL-8 secretion

**Figure 2:**
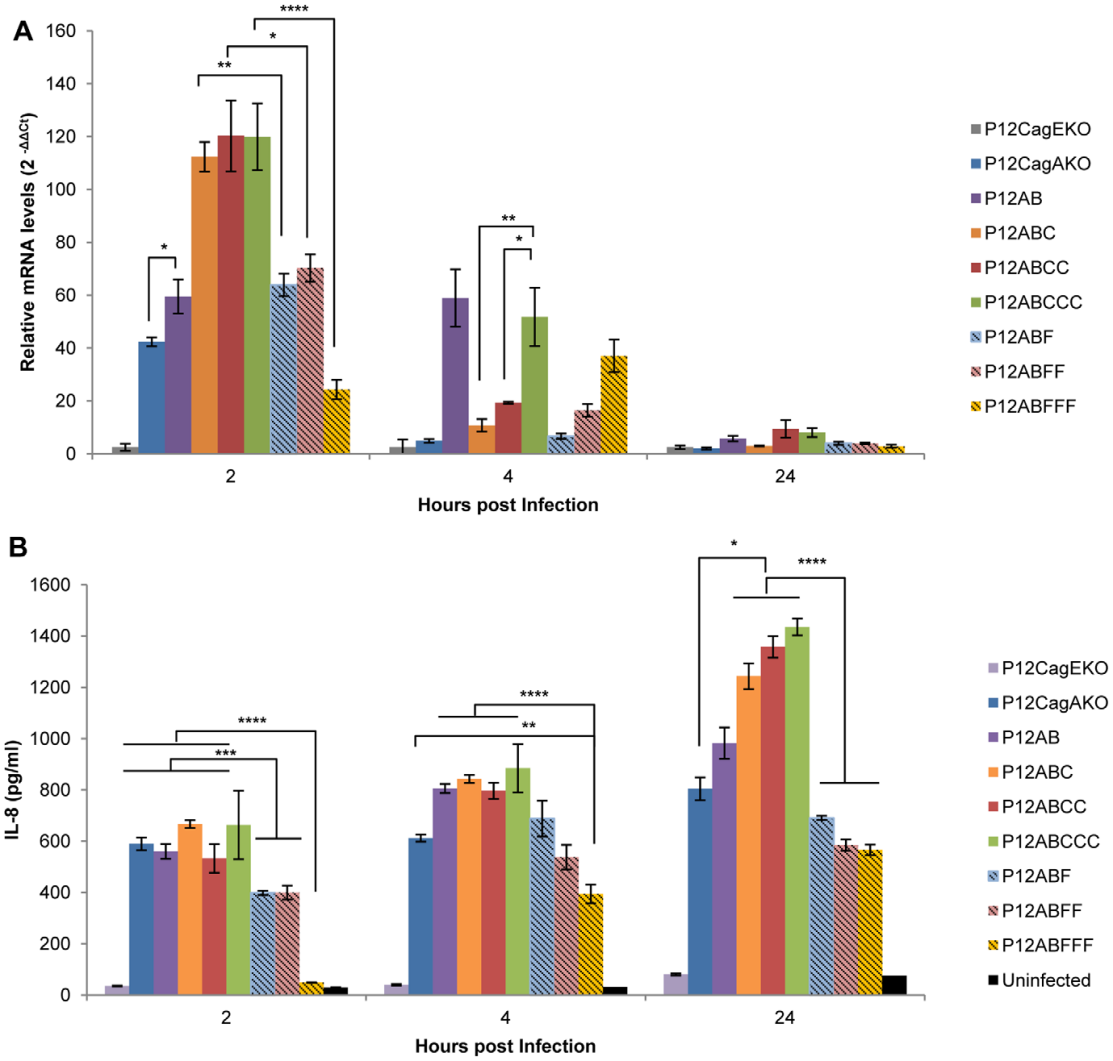
Effect of *H. pylori* infection on the induction and secretion of IL-8 by gastric epithelial cells. (A) Transcriptional activation of IL-8 gene in AGS cells, infected by the corresponding *H. pylori* mutant strains, expressing CagA with variable numbers of functional (EPIYA-C) or defective (EPIFA-C) phosphorylation motifs. IL-8 gene transcriptional activation was determined by a Quantitative Real Time PCR at 2, 4 and 24h post-infection. Statistical analysis was done by Student t-test and levels of significance depicted are *<0.05, **<0.01, ***<0.001, ****<0.0001. (B) IL-8 concentration levels determined by ELISA, in the supernatants of AGS cells, infected by *H. pylori* mutant strains. Statistical analysis and levels of significance as in (A).

In order to quantify potential effect of EPIYA-C phosphorylation on IL-8 induction we infected AGS cells with the whole range of mutant P12 strains, namely P12AB, P12ABC, P12ABCC (*wt*), P12ABCCC, P12ABF, P12ABFF, P12BFFF, P12CagEKO and P12CagAKO. Expression of CagA protein as well as EPIYA-C intracellular phosphorylation was observed at 2 hours post-infection (Figure 1C). IL-8 gene transcriptional activation was quantified at 2, 4, and 24 hours post-infection. Strains P12ABCCC, P12ABCC and P12ABC equally induced the highest levels of IL-8 transcription (120-, 120- and 112-fold respectively) compared to the uninfected control at 2 hours post-infection (Figure 2A). On the contrary, P12AB strain lacking the EPIYA-C phosphorylation sequences on CagA, induced IL-8 activation levels at 60-fold, a statistically significant reduction, compared to those induced by the strains P12ABCCC (*p* = 0.006), P12ABCC (*p* = 0.007) and P12ABC (*p* = 0.002). Moreover, IL-8 induced activation by the strains expressing CagA with phosphorylation-deficient EPIFA-C, was approximately halved for strains P12ABF and P12ABFF (64- and 70-fold) and even further reduced for the P12ABFFF (24-fold) strain at levels lower than those observed even for the P12CagAKO strain (42-fold). At 4 hours post-infection, IL-8 activation induced by the P12ABCCC strain was maintained at much higher level (50-fold) compared to those observed for P12ABCC (*p* = 0.001) and P12ABC strains (*p* = 0.0009, Figure 2A). These strains as well as P12CagAKO, P12ABFF and P12ABF induced background levels. Interestingly, IL-8 activation induced by P12AB strain remained unaltered between 2 and 4 hours, a result repeatedly obtained in over 5 repetitions of the experiment, for which we can offer no explanation. In contrast P12ABFFF strain contributed to a marginal increment between 2 and 4 hours (*p* = 0.05). At 24 hours post-infection IL-8 transcriptional activation in infected cells was at background levels for all strains (Figure 2A).

For the same experimental layout, we proceeded to determine IL-8 concentration in the supernatants of infected AGS gastric epithelial cell lines (Figure 2B). At 2 hours post-infection all strains induced similar amounts of IL-8 protein in the range of 600 pg/ml, with the exception of strains P12ABF or P12ABFF which exhibited significantly decreased levels (400 pg/ml) and P12ABFFF, for which levels of IL-8 protein were negligible and comparable to those observed for uninfected control cells (Figure 2B). Furthermore, P12ABFFF strain induced significantly lower levels of secreted IL-8 compared to all other strains, at 4 hours (*p* = 0.001) as well as 24 hours (*p* = 0.004) post-infection (Figure 2B). This characteristic delay in IL-8 secretion induced by strain P12ABFFF was apparent in all replicate experiments in which IL-8 levels secreted were found to be consistently lower than those observed even for the P12CagAKO strain. Similar results were obtained with another four independent P12ABFFF clones isolated during production (data not shown). At 24h all strains with functional EPIYA-C motifs (P12ABC, P12ABCC, P12ABCCC) induced approximately the same levels of IL-8 (Figure 2B), in any case significantly higher (*p* = 0.011) than those levels observed for the strain P12AB.

Collectively, these results suggest that phosphorylation of CagA in terminal EPIYA-C motifs contributes to IL-8 secretion by gastric epithelial cells. Most specifically, mutation of these EPIYA-C motifs, while keeping the CM motifs intact, leads to a characteristic delay in the initial transactivation of IL-8 gene, which consequently results to lower levels (approximately 2-fold reduction) of secreted IL-8, compared to the fully functional counterparts. Furthermore, secreted levels of IL-8 protein were observed not to be dependent upon the number of CagA EPIYA-C motifs, as similar levels were secreted by strains with one, two or three EPIYA-C motifs.

### NF-kB activation triggers the IL-8 induction with respect to EPIYA phosphorylation

**Figure 3:**
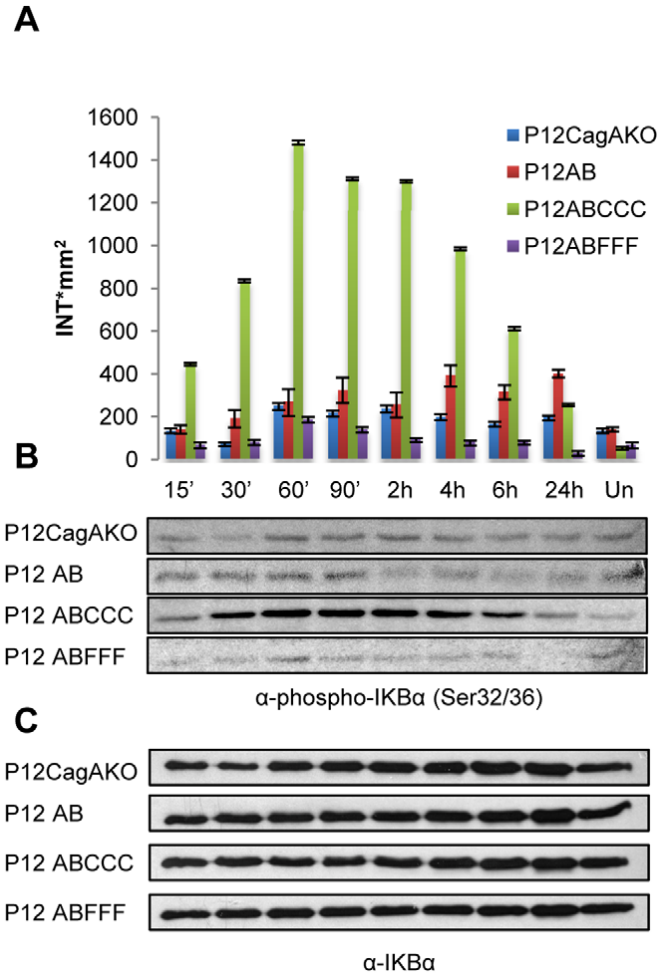
NF-kB activation in AGS cells following *H. pylori* infection. IkBα phosphorylation at Ser32/36 in AGS cells infected with *H. pylori* mutants, expressing CagA with phosphorylation-functional (EPIYA-C) or -defective (EPIFA-C) motifs. (A) Quantification of IkBα phosphorylation determined by band densitometry utilizing Quantity One software package. (B) Phosphorylation of IkBα and (C) Expression of total IkBα. Un: uninfected cells.

Induction of IL-8 following *H. pylori* infection of gastric epithelial cells has been attributed to NF-kB activation [9], [10], [30], [34], [44], [45]. In our study, we monitored NF-kB activation by determination of the kinetics of the Ser32/36 phosphorylation of its inhibitor IkBα, leading it to proteasome degradation [23]. As transcriptional activation and production of IL-8 proved to be independent of the number of EPIYA-C motifs, we proceeded to conduct the experiments utilizing P12ABCCC and the respective P12ABFFF mutant, as well as P12AB and the P12CagAKO strains. Following infection of AGS cells over 24 hours, P12ABCCC strain induced the highest phosphorylation of IkBα amongst all strains, in a time-dependent manner (Figure 3). Most specifically, increased IkBα phosphorylation was evident as early as 15 min post-infection, reaching a peak at about 1–1,5 hours and decreasing thereof to reach background levels at 24 hours. Concomitantly, a marginal decrease in IkBα expression levels was evident during the early stages post-infection (15–90 min) compared to the late ones (2–24 hours). Similar induction kinetics of IkBα phosphorylation was observed for strains P12CagAKO and P12AB, although levels were lower than those observed for P12ABCCC (Figure 3). On the contrary, strain P12ABFFF expressing CagA with phosphorylation-deficient motifs, exhibited the lowest IkBα phosphorylation levels while IkBα expression remained constant, throughout the experimental period (Figure 3). Collectively, these results illustrate that following infection of gastric epithelial cells with *H. pylori* strains, a higher activation of NF-kB is evident in the presence on phosphorylated EPIYA-C motifs. NF-kB is also activated, yet at considerably lower levels, in the absence of CagA expression or CagA phosphorylation and in the cases where EPIYA-C motifs are totally absent. Moreover, kinetics of IkBα phosphorylation (Figure 3) and hence NF-kB activation, appear to be in absolute agreement with the observed IL-8 transcriptional activation patterns (Figure 2A).

### ERK1/2 and AKT contribution to the activation of NF-kB following phosphorylation of CagA on EPIYA motifs

**Figure 4:**
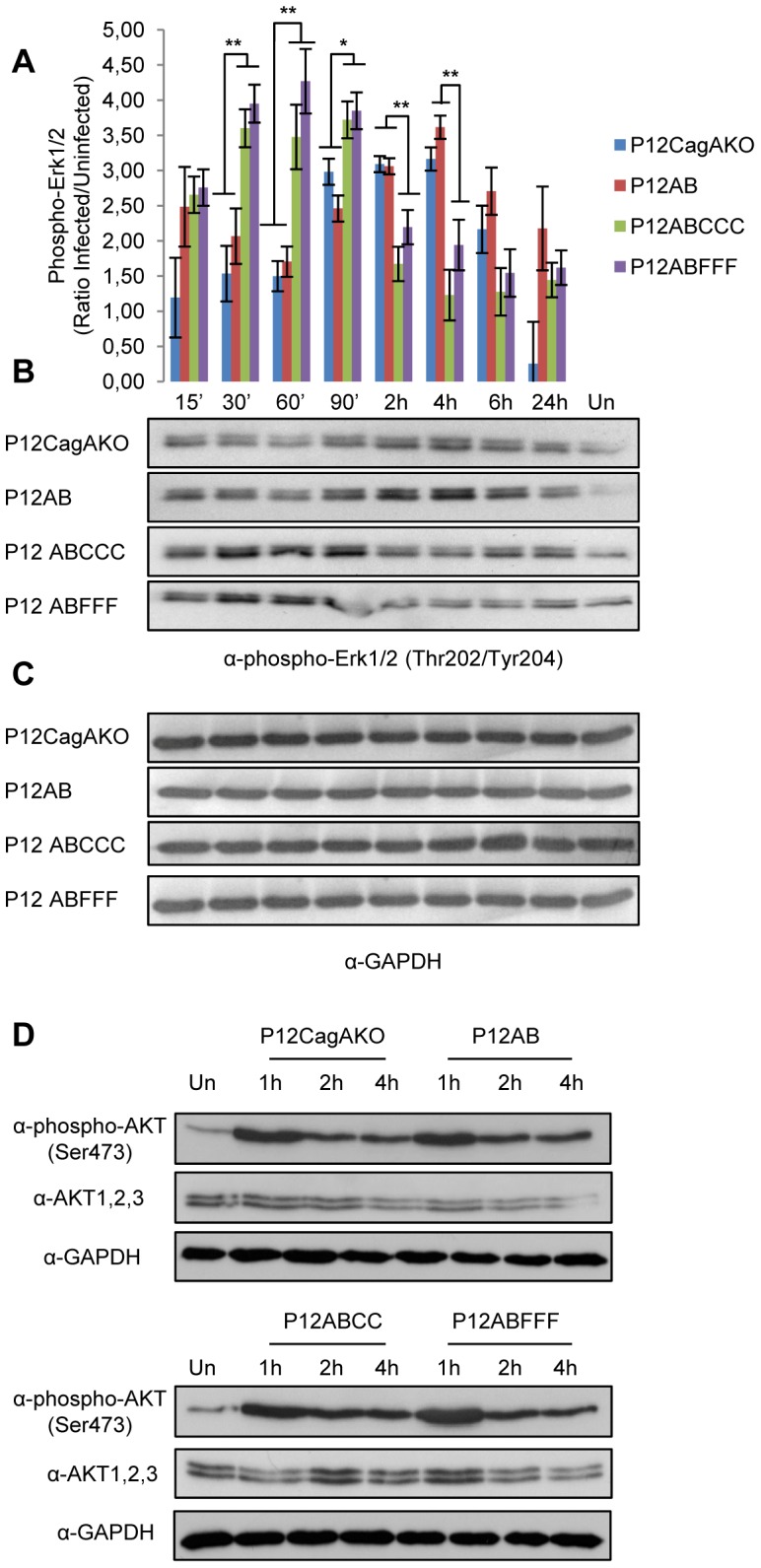
ERK1/2 and AKT activation in AGS cells upon infection with *H.* * pylori* **mutant strains expressing CagA with phosphorylation-functional (EPIYA-C) or -defective (EPIFA-C) motifs.** (A) Quantification of ERK1/2 phosphorylation at Thr202/Tyr204 by band densitometry in two independent experiments is depicted by plotting phospho-ERK1/2 (ratio infected to uninfected cells) for each time point.vStatistical analysis was done by Student t-test and levels of significance depicted are *<0.05 and **<0.01. (B) Representative ERK1/2 phosphorylation at Thr202/Tyr204 and (C) the corresponding GAPDH expression. (D) Phosphorylation of AKT at Ser473 determined in total protein lysates from AGS cells infected with corresponding AKT1, 2, 3 and GAPDH expression. Un: uninfected cells.

ERK1/2 activation in the CagA-dependent induction of IL-8 through NF-kB activation has been illustrated before [30]. In our study, we have monitored the kinetics of ERK1/2 activation by determination of phosphorylation at Thr202/Tyr204 [46]. Our results showed that in cells infected by P12ABCCC, as well as the corresponding P12ABFFF phosphorylation-deficient mutant, ERK1/2 activation occurred at similar significantly higher levels compared to strains P12AB and P12CagAKO, during the initial stages of infection (mean difference at 30 min of 1.97 and 95%CI 0.80 to 3.14 [*P* = 0.006], mean difference at 60 min of 1.06 and 95%CI of 0.27 to 1.84 [*P* = 0.004], mean difference at 90 min of 1.140 and 95%CI of 0.48 to 1.79 [*P* = 0.016]), suggesting that ERK1/2 activation is EPIYA-C motif phosphorylation-independent (Figure 4A and Figure 4B). On the contrary, strains P12AB and P12CagAKO induced similar significantly higher ERK1/2 activation levels much later, after the initial 90 min post-infection (mean difference at 2 hours of 1.80 and 95%CI of 0.83 to 2.77 [*P* = 0.005], mean difference at 4 hours of 1.03 and 95%CI -0.13 to 2.18 [*P* = 0.004]), suggesting that early ERK1/2 activation may depend upon the presence of sequences surrounding the EPIYA-C motifs. Recently, the CagA protein conserved amino acid sequence CRPIA was suggested to play a significant role in the activation of NF-kB via AKT kinase [31]. Our observations with regards to potential involvement of AKT in the activation of NF-kB suggest that the activation pattern of AKT does not match with this of NF-kB activation. More specifically, following *H. pylori* infection of AGS cells, we determined that activation of AKT Ser473-phosphorylation is independent of CagA EPIYA-C motif status (Figure 4D).

### Activation of NF-kB possibly through TAK1 activation following CagA EPIYA-C phosphorylation*v*

Recently, TAK1 was reported to be involved in *H. pylori*-induced NF-kB activation following its interaction with CagA protein and TRAF6 [34]. More specifically, it was suggested that CagA physically associates with TAK1 in vitro and in vivo and thereby enhances TAK1 activation which also requires TRAF6-mediated Lys63 ubiquitination. Furthermore, another study suggested that phosphorylated CagA can lead to activation of NF-kB and consequently transcriptional activation of IL-8, through p38 phosphorylation [47], whose upstream regulator is TAK1 [48], [49].

**Figure 5:**
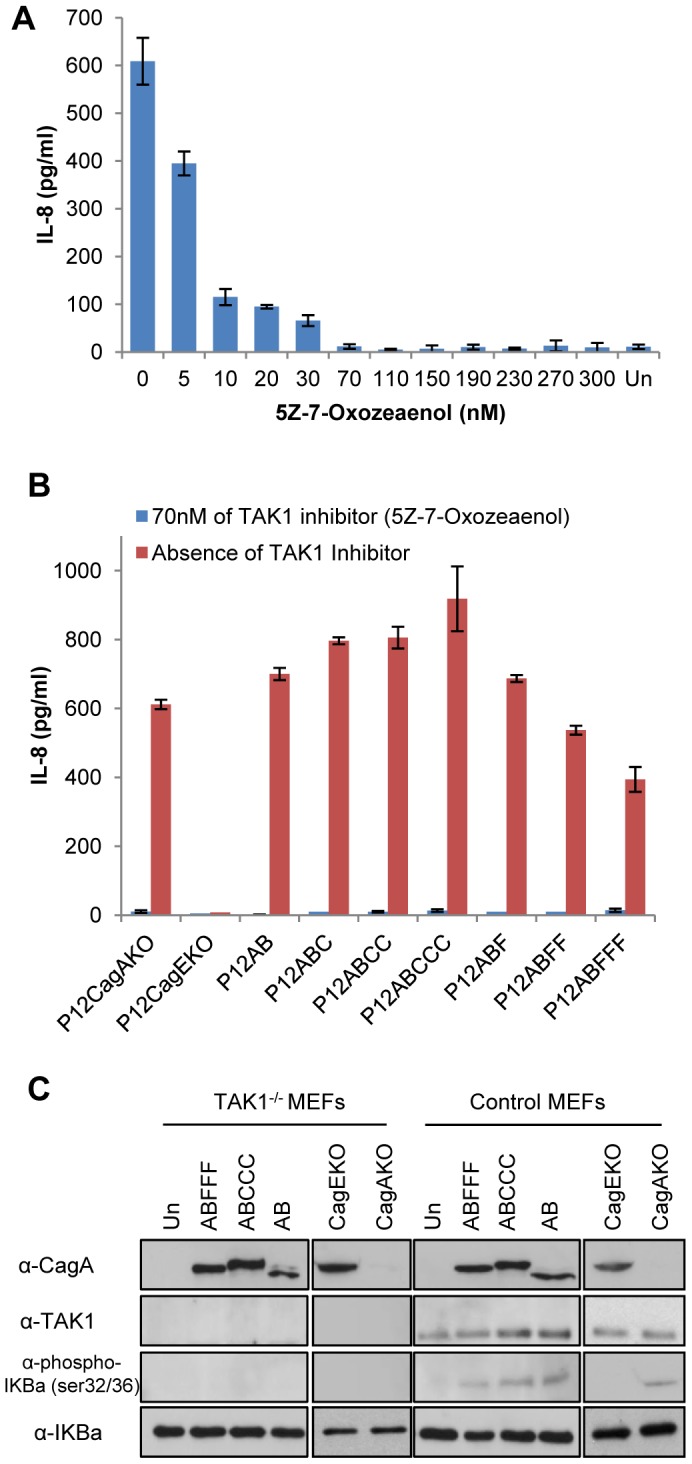
Potential involvement of TAK1 in IL-8 secretion following *H. pylori* infection. (A) Dose-dependent inhibition of IL-8 secretion, in the presence of the specific TAK1 inhibitor 5Z-7-Oxozeaenol (5–300 nM), following 4 hour infection of AGS cells with *H. pylori* P12ABCCC strain. (B) IL-8 secretion in the supernatants of AGS cells following 4 hour infection by the corresponding *H. pylori* CagA mutants, in the absence or presence of 70 nM 5Z-7-Oxozeaenol. (C) Expression of CagA, TAK1, IKBa phosphorylation at Ser32/36 and total IkBα determined by western blot, in total lysates of TAK1^-/-^ mouse embryonic fibroblasts (MEFs) and respective control MEFs infected with *H. pylori* mutant strains for 4 hours. Un: uninfected MEFs.

To confirm TAK1 involvement, we infected AGS cells with our CagA phosphorylation-functional and -deficient mutants in the presence of 5Z-7-Oxozeaenol which is reported to be a specific inhibitor of TAK1 in a number of studies [50]–[54] and determined the levels of secreted IL-8 in the culture supernatants (Figure 5A and Figure 5B), at 4h post infection, due to the labile nature of the inhibitor. Utilizing the P12ABCCC strain to infect AGS cells we observed that in the presence of TAK1 inhibitor IL-8 production was abrogated in a concentration-dependent manner (Figure 5A). Moreover, in the presence of 70 nM 5Z-7-Oxozeaenol we observed a complete arrest of IL-8 production by AGS cells infected with the whole range of CagA phosphorylation-functional as well as phosphorylation-deficient strains, P12CagAKO and the P12CagEKO (Figure 5B). In order to further clarify TAK1 involvement in *H. pylori* induced NF-kB activation we infected TAK1^-/-^ mouse embryonic fibroblasts (MEFs) and the respective normal control MEFs with P12ABCCC, P12ABFFF, P12AB, P12CagAKO and P12CagEKO mutants. We observed NF-kB activation following *H. pylori* infection, only in the presence of TAK1 expression in control MEFs and not in the TAK1^-/-^ cells (Figure 5C). As expected, unlike P12CagAKO which contributed to NF-kB activation in control MEFs, P12CagEKO did not induce NF-kB activation either in control or TAK1^-/-^ MEFs. Collectively these results suggest that NF-kB-dependent IL-8 induction in *H. pylori* infected gastric epithelial cells may possibly be mediated altogether through TAK1, irrespective of the CagA protein status.

**Figure 6:**
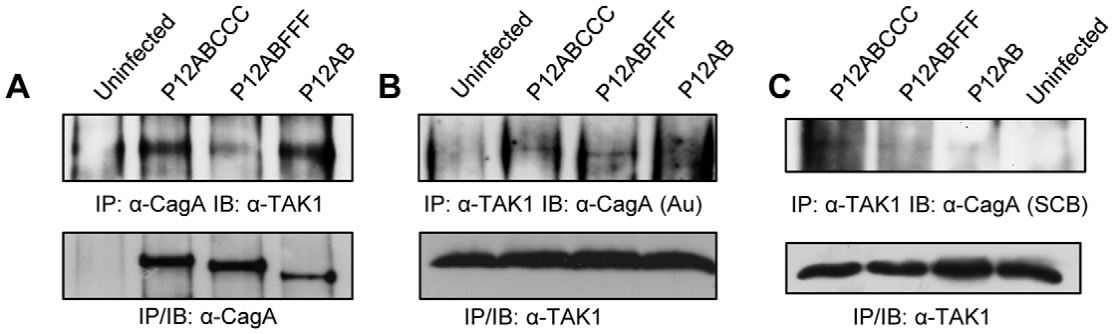
Immuno-detection of TAK1 and CagA in α-CagA or α-TAK1 respective immuno-precipitated lysates derived from AGS cells infected with *H.* * pylori* **CagA mutant strains, at 1 hour post infection.** (Panel A) Immunoprecipitation utilizing polyclonal α-CagA antibody (Austral Biologicals) and TAK1 western blot immunodetection utilizing monoclonal α-TAK1 antibody. Immunoprecipitation utilizing monoclonal α-TAK1 antibody and CagA immunodetection utilizing an α-CagA monoclonal antibody raised against the C-terminal (Panel B) or the N-terminal (Panel C) end of CagA protein. Note that the α-CagA monoclonal antibody raised against the C-terminal (Austral Biologicals) maps exactly on the EPIYA-C domains and fails to detect the P12AB CagA variant in panel B.

We proceeded to confirm by immunoprecipitation earlier observations which suggest that CagA may interact with TAK1 [34], in total lysates of AGS cells infected with the P12ABCCC, P12ABFFF and P12AB strains. We were able to identify TAK1 (Figure 6A) in anti-CagA immunoprecipitated protein complexes in total lysates of AGS cells infected with all three strains (P12ABCCC, P12ABFFF and P12AB).vEqually we were able to detect CagA proteins utilizing a C-terminal specific CagA antibody (Figure 6B), as well as, an N-terminal specific anti-CagA antibody (Figure 6C) within the monoclonal TAK1 antibody-immunoprecipitated protein complex. Our results suggest that CagA may participate in the same protein complex along with TAK1, irrespective of the presence or the phosphorylation status of EPIYA-C motifs in CagA. We also observed that following α-TAK1 immunoprecipitation, the α-CagA monoclonal antibody raised against the C-terminal (Austral Biologicals) failed to detect the P12AB CagA variant (Figure 6B) possibly because it maps exactly on the EPIYA-C domains. On the contrary, utilizing an N-terminal specific anti-CagA antibody (Figure 6C) we were able to detect CagA, suggesting that sequences following immediately after the EPIYA-C motif, such as the CM motif present in the P12AB CagA protein (Figure 1A and Figure 1B) may also possibly be implicated in the CagA-TAK1 interaction. This motif has been suggested to be a putative kinase anchor [33], however, further work is required to demonstrate this in our case. Finally, in order to clarify potential role of CagA protein EPIYA-C phosphorylation on TAK1 activation, we attempted to determine TAK1 phosphorylation on Thr187 as well as TAK1 protein expression in *H. pylori* infected AGS cells. However, we detected a very marginal increase in pThr187-TAK1 in P12ABCCC strain infected AGS cells, at 60 min post infection, compared to that observed in uninfected cells, followed by a decrease thereof (Figure S1A and S1B). Similar results were also observed for the P12ABFFF and P12AB strain-infected AGS cells, delayed for two hours, whereas no such TAK1 activation could be detected for cells infected with P12CagAKO strain. Such weak observations have been described before [34], with an equally weak signal for activated TAK1, a problem that is also presented in other studies [55].

## Discussion

*H. pylori* induces activation of a complex cytokine and chemokine network in the gastric mucosa and different bacterial and environmental factors, as well as host genetics seem to dictate the balance between tolerance and pro-inflammatory response in the course of *H. pylori* infection. CagA protein has been suggested to manifest its pathogenic role through EPIYA-dependent and EPIYA-independent interactions, in a number of intracellular signaling pathways, regulating cell motility and elongation, disruption of intercellular junctions and cellular polarity, as well as proliferation and inflammation mechanisms invoked by gastric epithelial cells [11]. Moreover, since most of these pathways have been implicated in the development of cellular malignancy there is increasing support that CagA protein, under persistent *H. pylori* infection may function as a bacterial oncoprotein [56], [57]. Presence of chronic active inflammatory response in the lamina propria is the hallmark of *H. pylori* infection and the main regulators of this inflammatory response are chemokines. *H. pylori* stimulates the gastric epithelium to produce IL-8, a potent neutrophil chemokine [5] and its activity was shown to correlate to histological severity in cases of *H. pylori*-induced antral gastritis [7]. Although early reports suggested that cagPAI functionality was a prerequisite for IL-8 induction and secretion by gastric epithelial cells [58], later studies proved beyond doubt, that this phenomenon was also potentiated by the expression of CagA protein [30]. Moreover, others suggested that IL-8 activation was not dependent upon the phosphorylation on EPIYA-C motifs but on a highly conserved amino acid sequence around EPIYA termed as CRPIA [31], also recognized to be a CagA multimerization motif [32], implicated in kinase anchoring and inhibition [33]. Very recently, *H. pylori* has been shown to induce IL-8 via interaction of its T4SS constituent CagL with the host receptor integrin b1 and the subsequent activation of MAPKs and NF-kB [36]. In this study we report that CagA phosphorylation on EPIYA-C motifs contributes to the induction of IL-8 gene in gastric epithelial cells, through activation of the NF-kB pathway, possibly via TAK1 interaction. Given the potential diversity of clinical strains, we utilized isogenic *H. pylori* CagA mutants based on the reference *H. pylori* strain P12, expressing CagA protein with variable numbers of EPIYA-C phosphorylation motifs (P12AB, P12ABC, P12ABCC, P12ABCCC) and their respective EPIFA-C phosphorylation deficient counterparts (P12ABF, P12ABFF, P12ABFFF), while maintaining intact the EPIYA-A and B motifs[37]. We carefully screened our *H. pylori* mutant strains with regards to growth rates, adhesion rates to gastric epithelial cells and for polar effects to other cagPAI genes, pilus formation, type IV secretion system functionality, as well as CagA expression and phosphorylation[37]. We demonstrated that induction of the scattering and elongation phenotype, was as expected, dependent upon the number of motifs and phosphorylation status of the EPIYA-C motifs[37]. A number of previous reports have been based upon observations from transfected CagA-expressing species which, although undoubtedly very valuable for the study of bacterial virulence factors, may lead to erroneous results due to potentially ectopic over-expression of the bacterial protein under study. Furthermore, confusing results due to individual strain genetic variability, have also been attributed to the use of clinical isolates expressing CagA with differential number of EPIYA-C motifs [35]. In our study, IL-8 induction and secreted levels do not appear to be dependent upon the number of EPIYA-C repeats within CagA, as all our strains with functional EPIYA-C motifs were capable of inducing the same levels of IL-8 transcriptional activity and chemokine. These results on IL-8 secretion, are in line with previous observations reported by our group utilizing 4 different pairs of isogenic clinical strains isolated from the same patient, expressing CagA with variable numbers of EPIYA-C motifs [27], [28]. In the same studies, CagA-positive clinical isolates with higher numbers of EPIYA-C motifs were not found to be associated with the severity of inflammatory response in the patient lamina propria. On the other hand, presence of phosphorylation-functional EPIYA-C motifs seems to contribute significantly to the *cagA*-dependent IL-8 induction, because deletion of these motifs, as in the case of P12AB, or mutation to the phospho-deficient EPIFA, in P12ABF and P12ABFF strains, dramatically reduced the levels of IL-8 induction. Most intriguingly, all P12ABFFF clones showed a characteristic delay in early IL-8 induction compared to all the other mutants, consistent of a potential transient inhibition of the T4SS, although earlier work has proven successful pilus formation and CagA translocation[37]. Alternatively, such an inhibition in IL-8 induction by the P12ABFFF strains may be associated with the presence within the CagA carboxyl-terminal region of four repeats of the putative kinase inhibitor motif FPLKRHDKVDDLSK [33], in the absence of any EPIYA-C functional domains, which dictate the hierarchic phosphorylation-dependent CagA activity [24].

In addition to the EPIYA-C effect, our data suggest that presence of EPIYA-A and EPIYA-B may marginally also contribute to IL-8 induction, because the CagA P12AB strain was able to induce significantly higher levels of IL-8 transcriptional activation compared to the P12CagAKO, yet similar to those observed following infection with the P12ABF and ABFF strains. This may also suggest that the number of CM does not seem to play a role in the induction of IL-8, as AB, ABF and ABFF have 1, 2 or 3 such motifs, respectively, and they all induce equal levels of IL-8 transcription at 2 hours, as well as NF-kB activation (data not shown). Furthermore, our results following infection with the P12CagAKO strain, are in line with observations suggesting *cagA*-independent IL-8 gene transcriptional activation and secretion, following recognition of peptidoglycans, by the intracellular Nod1 receptor [29] or *H. pylori* lipopolysaccharide by TLRs [45], [59] or CagL binding to integrin b1 [36]. In any case, our data suggest that *cagA*-dependent contribution through EPIYA-C phosphorylation may be equally significant for full activation of IL-8 transcriptional activity.

Another aim of this work was to shed light on the molecular pathway by which NF-kB is activated following EPIYA-C phosphorylation. Until recently, activation of NF-kB following *H. pylori* infection was shown to be a result of ERK and AKT activation [14], [30], [31]. Here we provide evidence that activation of ERK and AKT during the early stages post-infection may be influenced by the presence of EPIYA-C motifs, albeit in a phosphorylation-independent manner. In addition, our data suggest that activation of ERK and AKT does not exactly coincide to NF-kB activation. More specifically, we observed that in the presence of sequences surrounding EPIYA-C motifs, as in the case of P12ABCCC and P12ABFFF strains, induction of ERK1/2 was evident at early stages as quickly as 30’ post-infection irrespective of EPIYA phosphorylation. On the contrary, in the absence of EPIYA-C motifs as in the P12AB and P12CagAKO strains, ERK1/2 activation was evident at much later stages post-infection (2 - 4h). In the case of AKT, we observed a CagA-independent effect of *H. pylori* infection on AKT phosphorylation at Ser473. That is most likely consistent with its contribution to the control of survival and apoptosis, rather than that of other signaling pathways [60]. Further work needs to be done in order to understand how phosphorylation of CagA in EPIYA-C motifs may contribute to the activation of these two key molecules.

Finally, our data suggest that TAK1 may indeed play a critical central role in the activation of IL-8, because infection of gastric epithelial cells with all our *H. pylori* CagA mutants, in the presence of a TAK1 specific inhibitor [50]-[54], totally abrogated IL-8 secretion, even in the case of the P12CagAKO. Identical results were obtained when we infected TAK1^-/-^ mouse embryonic fibroblasts with our *H. pylori* CagA mutant strains including the CagA negative mutant and observed NF-kB activation only in the presence of TAK1 expression in control MEFs and not in the TAK1^-/-^ cells (Figure 5C). This suggests that TAK1 may be the converging molecule playing a central role for NF-kB activation during *H. pylori* infection, involving as well pathways activated by TLRs or NOD1 receptors as recently proposed [13]. Moreover, our data suggest that in the case of CagA-positive strains, TAK1 and CagA may participate in the same protein complex, confirming earlier observations showing that CagA can directly interact with TAK1 and TRAF6 [34]. Our results suggest that this TAK1-CagA interaction is independent of EPIYA-C-phosphorylation and may involve the CM motif preceding the EPIYA-C motif. Nevertheless, a whole range of experiments involving protein-protein interactions and mutational analysis needs to be performed in order to further dissect this interaction. We speculate that within the first hour post-infection, TAK1 may be recruited on CagA irrespective of EPIYA-C phosphorylation, however its consequent activation may depend upon EPIYA-C phosphorylation by c-Src and therefore TAK1 may be activated much faster in the presence of CagA-positive compared to CagA-negative strains. A possible explanation is that the different types of EPIYA phosphorylation motifs become phosphorylated by cellular kinases Abl and Src, at different time points following the delivery of CagA by the type IV secretion system. Indeed, EPIYA-C motifs are the first to be phosphorylated by Src, followed by Abl phosphorylation of EPIYA-A or EPIYA-B motifs [24], although more work is needed in order to explore this phenomenon with strains expressing CagA with multiple EPIYA-C motifs. Our results however, failed to show convincing TAK1 activation in the presence of functional EPIYA-C motifs in CagA compared to other CagA species examined in this study and further investigation is needed in order to prove this argument.

In conclusion, presence of functional EPIYA-C motifs in CagA protein, seem to contribute significantly in the transcriptional activation of IL-8, through NF-kB activation. This effect looks to be independent from *H. pylori*-dependent activation of AKT and ERK and may involve TAK1. This is the first systematic investigation of the role of EPIYA-C motifs in CagA in an isogenic background. Previous reports investigating their role in inflammation and disease outcome are based on comparison of different isolates. These comparisons however, suffer from the fact that *H. pylori* is genetically variable and has other phase-variable and mosaic genes. On the contrary, this is a systematic investigation and does not suffer from the aforementioned problems.

## Supporting Information

Figure S1 **TAK1 activation following infection of AGS cells with** ***H. pylori*** **CagA mutant strains.** (A) Quantification of TAK1 phosphorylation at Thr187 by band densitometry. (B) Expression of phospho-TAK1 (Thr187) in total protein lysates and corresponding (C) TAK1 protein expression and (D) control GAPDH expression in total protein lysates from AGS cells infected with *H. pylori* CagA mutant strains as indicated.
(TIF)
