# Supplementary figures and images for: Presence of Terminal EPIYA Phosphorylation Motifs in Helicobacter pylori CagA Contributes to IL-8 Secretion, Irrespective of the Number of Repeats
Source: PLoS One. 2013 Feb 7;8(2):e56291. doi: 10.1371/journal.pone.0056291 (PMC3567036; doi:10.1371/journal.pone.0056291)

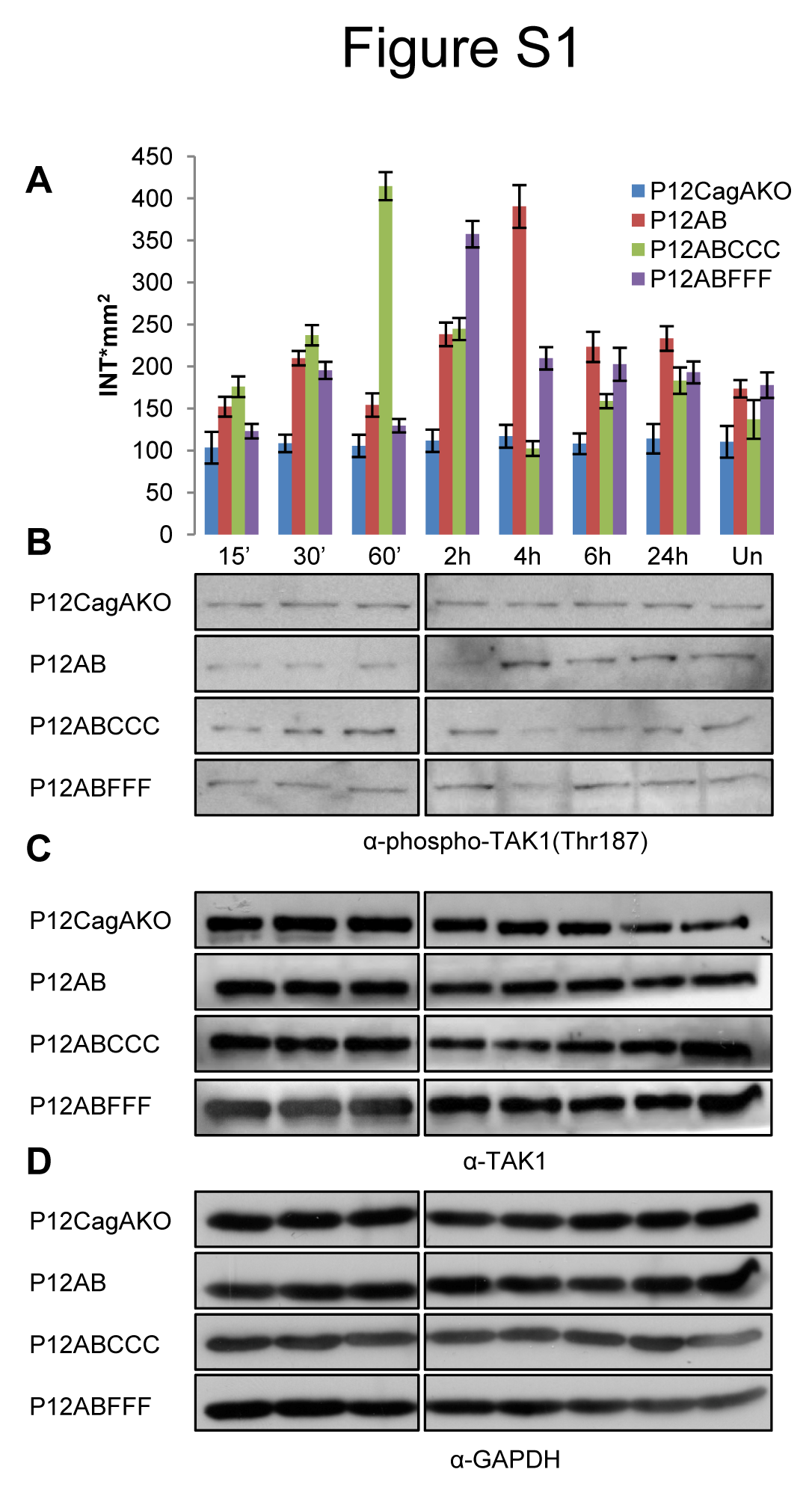

Supplement: Figure S1 — TAK1 activation following infection of AGS cells with H. pylori CagA mutant strains. (A) Quantification of TAK1 phosphorylation at Thr187 by band densitometry. (B) Expression of phospho-TAK1 (Thr187) in total protein lysates and corresponding (C) TAK1 protein expression and (D) control GAPDH expression in total protein lysates from AGS cells infected with H. pylori CagA mutant strains as indicated. (TIF) [file pone.0056291.s001.tif]
